# Precise Gene Knock‐In Tools with Minimized Risk of DSBs: A Trend for Gene Manipulation

**DOI:** 10.1002/advs.202401797

**Published:** 2024-05-10

**Authors:** Yongfeng Liu, Jianping Kong, Gongyu Liu, Zhaoxing Li, Yibei Xiao

**Affiliations:** ^1^ Department of Pharmacology School of Pharmacy China Pharmaceutical University Nanjing 210009 China; ^2^ State Key Laboratory of Natural Medicines China Pharmaceutical University Nanjing 210009 China; ^3^ Mudi Meng Honors College China Pharmaceutical University Nanjing 210009 China; ^4^ Chongqing Innovation Institute of China Pharmaceutical University Chongqing 401135 China

**Keywords:** DSBs, gene therapy, knock in, large fragment

## Abstract

Gene knock‐in refers to the insertion of exogenous functional genes into a target genome to achieve continuous expression. Currently, most knock‐in tools are based on site‐directed nucleases, which can induce double‐strand breaks (DSBs) at the target, following which the designed donors carrying functional genes can be inserted via the endogenous gene repair pathway. The size of donor genes is limited by the characteristics of gene repair, and the DSBs induce risks like genotoxicity. New generation tools, such as prime editing, transposase, and integrase, can insert larger gene fragments while minimizing or eliminating the risk of DSBs, opening new avenues in the development of animal models and gene therapy. However, the elimination of off‐target events and the production of delivery carriers with precise requirements remain challenging, restricting the application of the current knock‐in treatments to mainly in vitro settings. Here, a comprehensive review of the knock‐in tools that do not/minimally rely on DSBs and use other mechanisms is provided. Moreover, the challenges and recent advances of in vivo knock‐in treatments in terms of the therapeutic process is discussed. Collectively, the new generation of DSBs‐minimizing and large‐fragment knock‐in tools has revolutionized the field of gene editing, from basic research to clinical treatment.

## Graphical abstract

Gene knock‐in is one of the most popular focused technologies nowadays, and traditional knock‐in tools pose risks such as off‐target and genotoxicity due to induced double‐strand breaks (DSBs). The new generation of knock‐in tools such as PE, transposase, and integrase can not only minimize the risk of DSBs but also increase the load of insertion cargo, expanding its application.

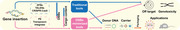

## 1 Introduction

Gene‐editing technologies that modify genes at specific sites have revolutionized bioscience, propelling advancements in basic research and showing immense potential for clinical therapy.^[^ ^1^ ^]^ A few key parameters, such as programmability, safety, and efficiency, are typically evaluated to develop or enhance these gene‐editing tools. Therefore, long‐term goals in this field include improving specificity, minimizing unexpected byproducts, establishing broad applicability across diverse organisms, and maximizing tool effectiveness.^[^ ^2^ ^]^

**Figure 1:**
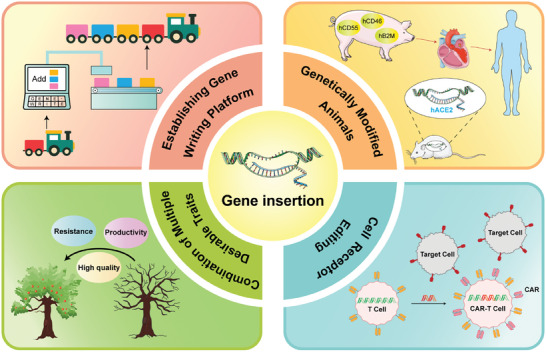
Applications and Prospects of Gene Knock‐in Technology. Gene knock‐in is used to modify genes for constructing animal models or achieving allogeneic organ transplantation in biomedical research.^[^ ^130^ ^]^ (Genetically Modified Animals) What's more, the engineering of cell receptors, such as the insertion of CAR (chimeric antigen receptor) genes to increase their ability to recognize and kill tumor cells, has opened up new possibilities for cancer treatments.^[^ ^125b^ ^]^ (Cell Receptor Editing) High‐yielding, resilient, nutrient‐rich plants have great economic value, which could be achieved by multiple orthogonal insertions.^[^ ^35^ ^]^ (Combination of Multiple Desirable Traits) The ultimate goal of gene insertion, achieving a gene writing platform, involves transforming target cells or organisms in a manner akin to synthetic biology concepts. (Establishment gene writing platform). Parts of the figure were drawn by using pictures from Servier Medical Art. Servier Medical Art by Servier is licensed under a Creative Commons Attribution 3.0 Unported License (https://creativecommons.org/licenses/by/3.0/). CAR (chimeric antigen receptor): An artificially modified receptor that could give immune cells the specificity to be activated by a specific target, thereby enhancing the ability of cells to recognize antigen signals and activation.

Despite advancements in gene‐manipulation techniques, including gene knockouts, gene silencing, gene expression activation, and base substitutions,^[^ ^3^ ^]^ the precise and programmatic insertion of large DNA sequences remains a prominent challenge. Gene insertion stands out for its critical roles in validating gene function and constructing disease models, thereby becoming an indispensable tool in drug discovery.^[^ ^4^ ^]^ Additionally, its applications extend to disease treatment by replacing defective genes or introducing functional copies, underscoring its versatility (**Figure** **1**).^[^ ^5^ ^]^ Compared with plasmids and other expression vectors, gene insertion offers distinct advantages, including lower immunogenicity, improved control over gene expression, enhanced safety, reduced metabolic burden on host cells, and more stable expression. For example, the in vivo expression efficiency of plasmid expression vectors is low, and the quality and stability of their expression are affected by their topology.^[^ ^6^ ^]^ In addition, the low transfection efficiency of plasmid expression vectors hinders their applicability.^[^ ^6^ ^]^ These benefits make gene insertion a preferred method for both research and therapeutic applications.

Conventional insertion tools are often limited by various issues. While employing modified viral systems to encapsulate target genes could ensure stable gene insertion, the randomness, and heterogeneity of these insertions into the human genome present serious safety issues.^[^ ^7^ ^]^ Unassisted homology‐directed recombination (HDR) displays exceptional targeting capability for inserting donor DNA but has low efficiency.^[^ ^8^ ^]^ Some evidence indicates that overexpressing genes that enhance HDR could boost knock‐in efficiency (discussed below).^[^ ^9^ ^]^ Site‐directed nucleases (SDNs), exemplified by clustered regularly interspaced short palindromic repeats (CRISPR), strike a balance between specificity and efficiency.^[^ ^10^ ^]^ These nucleases could induce double‐strand breaks (DSBs) at specific sites, significantly increasing the frequency of HDR.^[^ ^11^ ^]^ However, the reliance on DSBs might lead to off‐target events and severe genotoxicity, and the efficiency of these tools is still not satisfactory.^[^ ^12^ ^]^

Tools such as prime editing (PE) have been developed to minimize the drawbacks of DSBs.^[^ ^13^ ^]^ These tools effectively minimize off‐target effects, toxicity, and other defects, albeit with considerably limited cargo capacity. Several strategies have been undertaken to enhance the capacity of PE.^[^ ^14^ ^]^ It's also a trend to leverage and modify natural integration tools, such as integrases and transposons.^[^ ^15^ ^]^ Although these tools offer benefits such as large capacity and high activity, they necessitate the identification of specific landing sites to function effectively.^[^ ^16^ ^]^

The expansion of the gene knock‐in toolbox with minimized DSBs has advanced the field of gene therapy, with the potential to cure certain genetic diseases and even cancers. The upcoming discussion in this review prioritizes the introduction and evaluation of various gene knock‐in tools, particularly those that reduce the risks of DSBs (**Table** **1**). Furthermore, this review concludes the challenges in the application of gene knock‐in to in vivo therapy, summarizes the existing donor DNA and carriers, and evaluates the risks for off‐target editing. Finally, it discusses several attempts aimed at disease treatments.

**Table 1:** Summary of commonly used gene insertion tools.

Type	Name	Locator and Identify sites	Effector	Cargo capacity	Repair way	Off‐target	References
SDN	Meganuclease	MN Sites	Meganuclease	≈10 kb^a^ ^)^	HDR	+ + + +	[17]
	ZFN	ZF motif	Fok1 nuclease			+ + + +	[18]
	TALEN	TALEs repeat	Fok1 nuclease			+ + +	[19]
	HDR‐Cas9	Cas9/gRNA	Cas9 nuclease		HDR	+ + +	[20, 21]
	HITI‐Cas9		Cas9 nuclease		HITI	+ + +	[21a]
	vCas9		vCas9(Cas9 S55R+R976A+K1003A+T1314R)		MMEJ&HDR	+ + +	[27a]
	Cas12a	Cas12/gRNA	Cas12a (Cpf1) nuclease		HDR	+ +	[134]
	Cas12b		Cas12b nuclease			+ +	[135]
Prime editing	PE1	Cas9(H840A)/pegRNA	Cas9 H840A‐WT MMLV RT	<44 bp	unknown^c^ ^)^	‐	[13]
	PE2	Cas9(H840A)/pegRNA	Cas9 H840A‐MMLV RT mutant^b^ ^)^			‐	[13]
	PE3	Cas9(H840A)/pegRNA&nCas9/gRNA	Cas9 H840A‐MMLV RT mutant+ Cas9 D10A			+	[13, 14]
	PE4	Cas9(H840A)/pegRNA	Cas9 H840A‐MMLV RT mutant+ MLH1 dn			‐	[42a]
	PE5	Cas9(H840A)/pegRNA&nCas9/gRNA	Cas9 H840A‐MMLV RT mutant+ Cas9 D10A+ MLH1 dn			+	[42a]
	aPE	Cas9(H840A)/apegRNA^d^ ^)^	Cas9 H840A‐MMLV RT mutant			‐	[40]
	GRAND	Cas9(H840A)/pegRNA*2	Cas9 H840A‐MMLV RT	20–250 bp up to 1 kb		+	[46]
	twinPE	Cas9(H840A)/pegRNA*2&attB	Cas9 H840A‐MMLV RT+ Bxb 1	>7 kb		+	[47a]
	PASTE PrimeRoot niCPE	Cas9(H840A)/pegRNA*2&attB Cas9(H840A)/epegRNA*2&RS nCas12a/cicular RNA	Cas9 H840A‐RT L139P‐Bxb1 Cas9 H840A‐MMLV RT + Cre LbCas12a D156R+R1138A‐MMLV ‐RTΔRNase H	>36 kb^e^ ^)^ ≈11.1 kb ≈20 bp		+ + +	[47, 48, 49]
Tyrosine integrases	Cre‐loxP	loxP	Cre integrase	≈5.1 kb	‐	+	[82b]
	λint	attP attB	λint integrase	≈9 kb	‐	+	[76]
Serine integrases	Bxb1	attP attB	Bxb1 integrase	>36 kb^e^ ^)^	‐	+	[82b]
	φC31	attP attB	φC31 integrase	>100 kb^f^ ^)^	‐	+	[83a]
Transposon	SB	AT‐rich palindromic sequence (structure twistable)	SB transposase	>100 kb	‐	+ + +	[52, 53]
	PB	AATT‐ characterized sequence	PB transposase	>100 kb	‐	+ + +	[53, 57]
	Tol2	Weak consensus sequence	Tol2 transposase	>100 kb	‐	+ + +	[53, 60]
	VchCAST	Cascade/gRNA	TnsA/B	>10 kb	‐	+	[136]
	ShCAST	Cas12k/gRNA	TnsB	>2.5 kb	‐	+	[66, 71]
	shHELIX	Cas12k/gRNA	TnsB‐Homing endonuclease	>10 kb	‐	+	[70]
Other Insertion tools	dCas9‐SSAP	dCas9/gRNA	SSAP	>1 kb	‐	+	[85]

^a)^ The cargo capacity of an editing system based on the DNA repair mechanism within the cell depends on the DNA carrying capacity of the delivery system used, and here we list a typical cargo capacity size.

^b)^ MMLV RT (D200N+L603W+T330P+T306K+W313F) is hereinafter referred to as MMLV RT.

^c)^ PE4 and PE5 effectively enhance the editing efficiency of PE by translating the dominant negative mutant (MLH1 dn) of MLH1, a key factor in the MMR repair pathway.

^d)^ Stability is improved by introducing more G‐C into pegRNA small hairpin structures.

^e)^ There is no obvious upper limit on the size of the donor DNA.

^f)^ Related to the distance between sites; inherent reaction bidirectionality.

## 2 Gene Insertion via the Cleave‐Repair Pathway

Gene insertion based on SDNs has been used extensively for a long time and has reached maturity. These platforms function by programming nucleases to cleave the desired DNA at specific sites, inducing DSBs, followed by insertion of donor DNA via host DNA‐repair pathways. Early‐stage tools such as homing endonucleases (also named meganucleases),^[^ ^17^ ^]^ zinc finger nucleases,^[^ ^18^ ^]^ and transcription activator‐like effector nucleases^[^ ^19^ ^]^ exhibit limited programmability and require complex protein‐DNA interactions for target identification. Since 2012, the advent and implementation of the CRISPR‐associated protein (Cas) system have resulted in ground‐breaking advancements.^[^ ^20^ ^]^ The nuclease Cas9 or Cas12 could bind to a single guide RNA (sgRNA) and then identify the target under the guidance of the sgRNA, via complementary base pairing.^[^ ^11a^ ^]^ This theoretically equips us with the capability to insert genes virtually anywhere, by designing the sgRNA.

**Figure 2:**
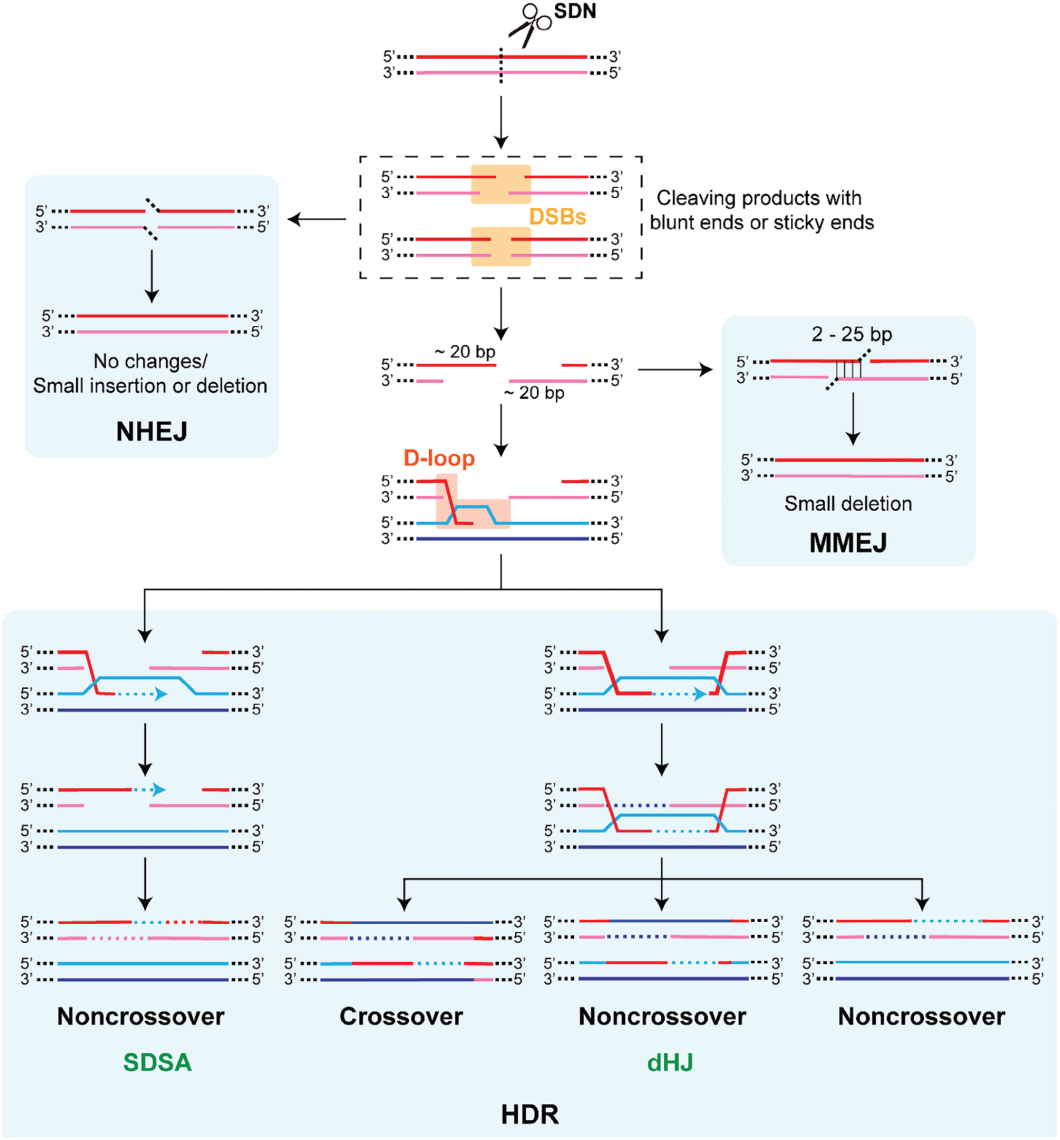
Three Major DNA Repair Pathways for Double‐Strand Breaks (DSBs). DSBs could be repaired by non‐homologous end joining (NHEJ), which rejoins the two ends of the DSBs. Compared with other alternative repair pathways, NHEJ has a lower probability of error, but it might still lead to the insertion or deletion (indels) of small DNA fragments.^[^ ^23^ ^]^ In fact, the risk of blunt‐end DSBs products is low, whereas sticky‐end DSBs products often introduce indels after repair via the NHEJ pathway. MMEJ uses a 2–25 bp microhomologous region on both sides of the DSBs to repair DNA.^[^ ^26^ ^]^ During partial resection of DNA ends, the microhomologous region is exposed, which then anneals. After flap trimming, a partially deleted product is finally formed. Homology‐directed repair (HDR) requires extensive homologous arms for homologous recombination, which makes it highly fidelity.^[^ ^8^, ^24^ ^]^ However, the activity of HDR is strictly regulated by cell cycle and DNA damage signals, and HDR occurs only in G2 and S phases (because under physiological conditions, sister chromosomes could only break away from histones at G2 and S phases and serve as templates for HDR).^[^ ^25^ ^]^ HDR could function by several pathways. Most non‐crossover gene conversion events are the results of an SDSA mechanism, whereas an alternative pathway leads to the formation of a pair of dHJ that could be resolved with or without crossover.^[^ ^132^ ^]^ SDNs (site‐directed nucleases): Enzymes that induce double‐strand breaks at specific DNA locations to initiate precise gene editing. SDSA (synthesis‐dependent strand annealing): A homologous recombination mechanism that repairs DSBs without altering chromosome structure, resulting in noncrossover outcomes. dHJ (double Holliday junction): An intermediate formed during homologous recombination that could be resolved into either crossover or noncrossover products, depending on the resolution pathway.

DSBs are the most cytotoxic of the possible breakages of DNA and are therefore strictly regulated in cells. SDNs‐based knock‐in tools make use of DSBs: the functional DNA is likely to be used to “repair” DSBs to achieve the purpose of insertion, and thus, such tools are highly dependent on the gene repair pathway in vivo.^[^ ^21^ ^]^ Owing to this, the activities of such knock‐in tools vary considerably, even within the same cell at different physiological stages. For instance, common in vivo repair pathways in eukaryotic cells include non‐homologous end joining (NHEJ), microhomology‐mediated end joining (MMEJ), and HDR (**Figure** **2**).^[^ ^22^ ^]^ NHEJ operates throughout the cell cycle, with a primary focus on rejoining broken double strands.^[^ ^23^ ^]^ Nonetheless, NHEJ lacks precision, often producing insertions or deletions of small fragments following NHEJ ligation.^[^ ^21^, ^23^ ^]^ In contrast, HDR is highly accurate, relying on the extensive homologous arms at both ends of the donor DNA (the length varies from dozens of bp to several kb in different conditions).^[^ ^8^, ^24^ ^]^ However, during most of the cell cycle, the efficiency of HDR is extremely low, which hinders its application.^[^ ^25^ ^]^ Combining the merits of HDR and NHEJ, MMEJ employs a microhomologous region (2–25 bp)^[^ ^26^ ^]^ on either side of DSBs, to repair DNA. The DNA ends are processed to expose the homologous sequence, followed by annealing and synthesis, before the final ligation that retains one of the microhomologous regions.^[^ ^26a^ ^]^ Even though the repair methodology of MMEJ is more intricate and less “perfect,” as compared with that of HDR, its repair product is highly predictable, as opposed to that of NHEJ.^[^ ^27^ ^]^ Furthermore, MMEJ also has a much higher frequency and active period than HDR, making it a promising repair pathway.^[^ ^22^ ^]^ Besides these repair pathways, other pathways like single‐strand annealing also demonstrate potential for gene insertion.^[^ ^21a^ ^]^

Numerous enhancements have been made to cleave‐repair gene‐insertion tools. The most effective of strategies involves fully exploiting the “competitive relationship” of intracellular repair pathways,^[^ ^28^ ^]^ which includes enhancing the efficiency of HDR repair while inhibiting “error‐prone” repair pathways such as NHEJ and MMEJ. This goal could be achieved via the single or combined use of small molecules or macromolecules and knockouts of key proteins in NHEJ and MMEJ.^[^ ^29^ ^]^ In addition, the structures and modifications of donor DNA greatly impact the efficiency of fragment knock‐in (discussed below). Furthermore, it is important to modify the nucleases for different scenarios. For instance, engineered nucleases that exhibit high activity even at low temperatures could fulfill the criteria for crop enhancement in specific situations.^[^ ^30^ ^]^ Moreover, sgRNA‐guided nucleases, such as Cas9 and Cas12, are also limited by the protospacer adjacent motif (PAM),^[^ ^31^ ^]^ thus making it difficult to target specific gene sites of interest. Utilizing nuclease mutants with more flexible PAM preferences will expand their applications.^[^ ^32^ ^]^

## 3 Precise Knock‐in with Minimized Risk of DSBs

### 3.1 PE

**Figure 3:**
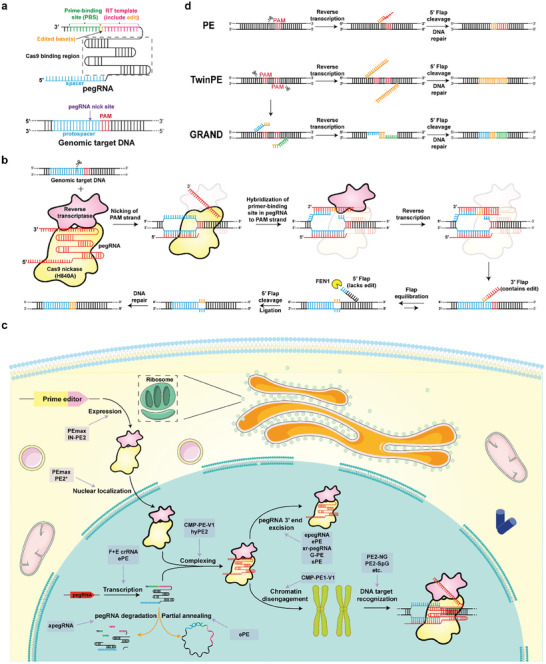
PE‐based Precise Gene Insertion. As a tool that minimizes the risk of DSBs, PE provides a safer and more accurate choice. a) Schematic diagram of the guideRNA of Prime editing (pegRNA) and its target DNA. Structural modificat. For example, adding more C‐G base pairs to the stem‐loop structure could make it more stable. Also, adding hairpin structures at the 3′ end could stop it from breaking down too soon. ions to pegRNA could enhance the efficiency of the PE system.^[^ ^13^ ^]^ b) Molecular mechanism of PE. Guided by pegRNA, the H840A‐Cas9 nickase (nCas9) cuts the strand of the target sequence that contains PAM, exposing its 3′ ends to bind with the pegRNA's PBS. Then, reverse transcriptase starts and edits using the pegRNA's RT template. After 3′ −5′ flap equilibration, the unedited 5′ flap is degraded by Flap endonuclease 1 (FEN1), and finally stably edited DNA is generated by ligation and DNA repair.^[^ ^13^, ^34^ ^]^ c) A schematic diagram of the workflow in vivo and variants of PE. PE‐Max, IN‐PE2 increase the expression of PE fusion protein. PE‐Max, PE2* enhances the nuclear localization ability of PE fusion protein.^[^ ^42^ ^]^ PE equipped with F+E crRNA and ePE could prevent the issue of pegRNA becoming circular.^[^ ^38^, ^39^ ^]^ This problem happens when the ends of pegRNA partially match each other. It is avoided by processing pegRNA with Csy4, which helps maintain editing efficiency.^[^ ^38^, ^39^ ^]^ aPE effectively reduces the degradation of pegRNA.^[^ ^40^ ^]^ CMP‐PE‐V1 and hyPE2 play important roles in the formation of protein‐pegRNA complexes.^[^ ^43^ ^]^ epegRNA, ePE, xr‐pegRNA, G‐PE, and sPE effectively prevent the degradation of the 3′ end of pegRNA.^[^ ^37^, ^41^, ^133^ ^]^ CMP‐PR‐V1 makes chromatin easier to open.^[^ ^43a^ ^]^ Cas9 mutants used by PE2‐NG and PE2‐SpG have less PAM restriction, making gene editing more widely available.^[^ ^42^, ^44^ ^]^ d) Overview of the characteristics of GRAND and Twin PE compared to PE. GRAND and twinPE are tools designed for gene insertion, differing from traditional PE. GRAND could insert up to 1 kb by using complementary flaps on both ends, overcoming the size limitations of PE and offering higher insertion efficiency for 20–250 bp.^[^ ^46^ ^]^ Meanwhile, twinPE could insert more than 7 kb by incorporating transposons.^[^ ^47^ ^]^ Parts of the figure were drawn by using pictures from Servier Medical Art. Servier Medical Art by Servier is licensed under a Creative Commons Attribution 3.0 Unported License (https://creativecommons.org/licenses/by/3.0/).

Given the complications associated with gene insertion via DNA‐repair mechanisms in response to DSBs, there is an urgent need for a DSBs‐minimized insertion toolkit. In 2019, Liu et al. made a breakthrough by discovering that a common mechanism known as 3′ flap‐5′ flap equilibration, instrumental in Okazaki fragment maturation and branch migration, could enable the insertion of desired genetic material into DNA duplexes, with fewer DSBs.^[^ ^13^ ^]^ This discovery led to the development of a new gene‐editing tool named prime editing 1 (PE1). PE1 consists of the H840A‐Cas9 nickase (nCas9), PE guide RNA (pegRNA, guide RNA containing a reverse transcription template and requisite prime binding site), and a wild‐type Moloney murine leukemia virus reverse transcriptase variant.^[^ ^33^ ^]^ The latter two components are linked via a loop structure (**Figure** **3**a). The process begins with nCas9 cleaving the PAM‐containing strand to generate a nick and expose the generated 3′ flap. Next, the PE‐pegRNA complex attaches to the 3′ flap though prime binding sites, thereby securing the insertion of the desired genetic information into the 3′ flap based on the pegRNA template. Separation of the fusion protein from the double‐stranded DNA (dsDNA) triggers the 3′ flap‐5′ flap equilibration mechanism, establishing a dynamic equilibrium between the flaps. Flap endonuclease 1 (FEN1), an essential host enzyme, cleaves the 5′ flap, shifting the equilibrium toward the 3′ flap binding to the dsDNA.^[^ ^34^ ^]^ Subsequently, the ligase repairs the nick to insert the edited flap into the targeted dsDNA. Lastly, DNA mismatch repair mechanisms repair the unedited strand, inserting the desired genetic information into the targeted gene locus (Figure 3b). As this technology reduces the DSBs events and doesn't need donor DNA, it offers a high safety profile in gene editing.^[^ ^35^ ^]^

However, the first generation of PE has limitations, such as poor thermal stability, weak processing ability, and low affinity for DNA‐RNA substrates of the reverse transcriptase used.^[^ ^36^ ^]^ The introduction of a modified reverse transcriptase into this system improved the efficiency of this editing tool.^[^ ^36^ ^]^ Another crucial concern is that the new strategy has a relatively lower editing efficiency compared to traditional gene‐insertion tools that generate DSBs.^[^ ^37^ ^]^ To address this shortcoming, many improvements have been made by considering various aspects of its in vivo editing cycles (Figure 3c). Strategies for improvement have included increasing the effective concentration of PE‐pegRNA;^[^ ^38^ ^]^ introducing a nuclease Csy4 recognition site into pegRNA, such as carrying F + E crRNA and ePE to help form pegRNA with the correct conformation;^[^ ^38^, ^39^ ^]^ engineering pegRNAs, such as apegRNA and spegRNA, to enhance their stability;^[^ ^40^ ^]^ designing the secondary structure of pegRNA to ensure that the exposed pegRNA 3′ end of the complex for editing is not degraded by endogenous substances;^[^ ^37^, ^41^ ^]^ and incorporating more signal peptides in the PE fusion protein for efficient entry into the nucleus.^[^ ^42^ ^]^ In addition, CMP‐PE‐V1 and hyPE2 improved the ability of effector proteins to form effector complexes with pegRNA in the nucleus.^[^ ^43^ ^]^ The former also has an improved chromosome unwinding ability, and therefore, stronger editing activity.^[^ ^43a^ ^]^ The dependence on PAM inherited by the Cas9 protein limits the unrestricted use of PE to some extent; PE2‐NG and PE2‐SpG have been developed to overcome this limitation and enable broader utilization of this tool.^[^ ^42^, ^44^ ^]^ The above PE‐based improvement tools could also be combined with each other to complement each of their strengths and weaknesses, thereby enhancing the ease of use of PE in the gene‐editing process, as well as its efficiency and safety.

Despite these improvements, lengthening the insert fragments remains a challenge for the existing PE modifications.^[^ ^45^ ^]^ Thus, PE insertion systems intended for improving the gene‐insertion capacity of PE for longer desired genetic information have been developed. One approach is to use a pair of pegRNAs, each targeting a different genomic location, thereby triggering widespread gene rearrangement between loci. An example is the GRAND gene‐editing system,^[^ ^46^ ^]^ which has shown remarkable insertion efficiency without requiring donor DNA or relying on homologous recombination enzymes for repair (Figure 3d). Another strategy combines a PE system with other gene‐insertion elements for two‐step insertion, such as the twin PE gene‐editing system.^[^ ^47^ ^]^

In summary, PE is a powerful gene‐editing tool capable of all types of base substitutions and gene additions/deletions with minimized‐DSBs, while maintaining a low off‐target rate. Although several PE variants have emerged based on different strategies, the orthogonality among these strategies remains unexplored. Interestingly, the recently reported novel PE composed of Cas12a and a circular guide RNA demonstrates more precise gene knock‐in and more freedom in terms of target selection, and thus, may indicate a new direction.^[^ ^48^ ^]^ The potential of PE for inserting larger segments, especially when coupled with other genetic components,^[^ ^47^, ^49^ ^]^ signifies valuable research prospects for expanding and improving PE‐compatible toolkit.

### 3.2 Transposons and Engineered Transposases

Transposons, or “jumping genes” are a type of mobile genetic element with diverse components and translocation strategies.^[^ ^50^ ^]^ Transposons fall into two categories: Class 1 retrotransposons (which depend on RNA intermediates) and Class 2 DNA transposons. Transposons could migrate and multiply within the host genome through replication, splicing, rolling cycles, and self‐replication.^[^ ^51^ ^]^ Their ability to implant large DNA fragments into target sequences makes them potential gene‐insertion tools. Despite their ubiquitous presence in nearly all eukaryotes, the utilization of natural transposons is limited by factors such as specificity, activity, and safety.^[^ ^51^ ^]^ A potential solution could be to screen transposon libraries for attributes such as target sequence specificity, enzyme engineering programmability, and safety of insertion preference using high‐throughput platforms.

**Figure 4:**
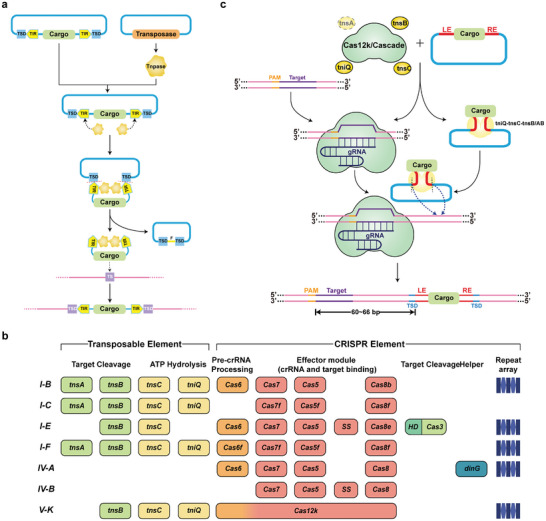
Large Fragment Gene Knock‐in Based on Transposition Mechanism. a) Overview of “cut‐paste” transposition. The transposase (Tnpase) binds to the terminal inverted repeats (TIRs) and induces DSBs, then removes the removable element from the donor DNA and leaves the footprint. The cargo genes are mobilized from donor DNA vectors by Tnpase to genomic sites. When the cargo‐transposase complex finds a suitable target site (TS), the cargo will be integrated and the target site duplication (TSD) will be produced.^[^ ^53^ ^]^ b) The current classification of CRISPR‐associated transposase (CAST) and its modular organization. Some subtypes lack CRISPR arrays and may use CRISPR arrays from other active CRISPR‐Cas systems. In addition, the transposable element of type IV CAST is unknown and it is speculated that it is a Tn7‐like transposon according to the cryo‐EM analysis of the type IV CAST effector complex. SS refers to the small that may be involved in the formation of Cascade (such as Cse2). The two colors of Cas12k indicate that it is involved in different stages of CRISPR‐Cas reaction.^[^ ^68^ ^]^ c) The mechanism of gene insertion in the classical CAST system. The guide RNA directs the single effect element Cas12k (PAM dependent) or Cascade (PAM independent) to the target site, recruits the transposable elements TniQ, TnsC, TnsB (or TnsA/B complex), and integrates the donor DNA fragment with the transposase recognition terminal at the position tens of bp downstream of the target site.^[^ ^69^ ^]^ Transposase (Tnpase): Enzyme that performs transposition function. It is usually encoded by the transposon and recognizes the specific sequences at both ends of the transposon. It could separate the transposon from the adjacent sequence and insert it into the new genomic site. TIR (terminal inverted repeat): A pair of short fragments with the same nucleotide sequence but opposite orientation exists at the end of some transposons. TSD (target site duplication): Two identical repetitive sequences formed from the target site after transposition, whose sequence reflects the specific target site preferences of every transposon family.

The most commonly used transposon systems include Sleeping Beauty (SB), PiggyBac (PB), and Tol2.^[^ ^52^ ^]^ These tools are based on the “cut‐paste” mechanism and typically consist of transposase genes and terminal inverted repeats (TIRs) that carry transposase binding sites at both ends. Transposases facilitate the activation of transposon cleavage (**Figure** **4**a),^[^ ^53^ ^]^ enabling the transposon TIR and transposase to be delivered separately through a two‐component carrier system. One carrier expresses a mobilizing transposase, while the other replaces the enzymatic sequence between the original TIRs and cargo genes. This allows for the potential incorporation of antibiotic resistance markers and reporter genes, making the system highly customizable. The principal advantages of this form of insertion tool include simplicity, relative safety, and large cargo capacity (up to over 100 kb).^[^ ^53^ ^]^ The SB system has shown promise for gene therapy due to its nearly random distribution of insertion sites (unlike PB and Tol2, which tend to favor proto‐oncogene‐rich regions) and its high transposition activity in difficult‐to‐transfect cells such as stem cells.^[^ ^54^ ^]^ Although the SB system has been widely used in chimeric antigen receptor T‐cell therapy,^[^ ^55^ ^]^ it should be noted that it is more prone to overproduction inhibition (a scenario wherein excess transposase expression hampers transposition activity) than the other systems.^[^ ^56^ ^]^ The activity of PB rivals that of SB. A unique and valuable feature of PB is its ability for traceless transposition, enabling untraceable editing.^[^ ^57^ ^]^ The molecular mechanism of traceless editing enables the precise excision of PB and TTAA overhangs at the 3′ and 5′ ends of the donor DNA following cleavage. These overhangs could easily be paired and linked through host repair mechanisms, resulting in TTAA sites at the donor site that are comparable to those at the original target site. Tol2 is advantageous because it could carry larger DNA fragments without compromising on the delivery efficiency.^[^ ^53^ ^]^ Moreover, Tol2 is virtually unaffected by overproduction inhibition and is extensively used in gene screening and the generation of transgenic animal lines.^[^ ^53^ ^]^

Strategies to increase the efficiency of transposon tools, including optimization and modification of TIRs, directed evolution of transposases, and innovation in delivery routes. Appropriate modifications to TIRs could enhance their affinity for transposase.^[^ ^58^ ^]^ In addition, truncations or mutations of TIR components may be used to boost the transposition efficiency under the premise of clarifying the function of the element.^[^ ^59^ ^]^ Another important approach is to augment the activity and expression of the transposase.^[^ ^60^ ^]^ For more efficient screening of mutants, a reasonable increase or substitution of amino acids could facilitate an increase in transposase activity. However, it must be noted that an increase in activity has not been observed across all cell lines, suggesting the need to select an appropriate transposase tool based on specific requirements. Retrotransposons have potential applications in high‐copy number transgenic cell lines, because of their self‐replicating properties. However, under physiological conditions, runaway proliferation of retrotransposons is closely associated with aging and cancer.^[^ ^61^ ^]^ Consequently, organisms strictly regulate retrotransposons. The potential risks limit the development of gene therapy tools. Future advancements will focus on achieving precise regulation and monitoring of these tools.

Transposon systems combined with CRISPR‐Cas have become the focus of gene‐insertion tool development, to overcome the limitations of weak targeting ability and low target programmability when using only a transposon system. Two primary approaches exist for the same: one involves the artificial Cas‐transposase fusion protein system,^[^ ^62^ ^]^ while the other involves the natural transposon system associated with CRISPR‐Cas. The CRISPR‐Cas system is generally believed to function as an acquired immune system in bacteria and archaea, providing defense against and removal of mobile genetic elements like exogenous transposons and phages.^[^ ^63^ ^]^ However, in reality, the relationship between the CRISPR‐Cas system and transposons is much more intricate. For instance, gene alignment analysis suggests that Cas1 in the CRISPR adapter bears high homology with many transposon‐encoded transposases, known as casposons.^[^ ^64^ ^]^ Furthermore, some viruses hijack parts of the CRISPR‐Cas system, such as the CRISPR array and the effector enzyme. While these hijacked elements lose their cleavage activity, they retain their RNA‐guided targeting activity, which may help them to recognize and compete with other viruses.^[^ ^65^ ^]^

Therefore, the CRISPR‐associated transposase (CAST) system was characterized and applied. Initially, Sternberg et al. and Zhang et al. characterized CASTs (shCAST and TnCAST) from *Vibrio cholerae* Tn6677 and *Scytonema hofmannii*, respectively.^[^ ^66^ ^]^ Subsequently, new CASTs were continuously predicted and characterized (Figure 4b).^[^ ^67^ ^]^ A classical CAST (type I and type V) primarily comprises three parts: i) CRISPR‐Cas element, Cas12k or cascade, and guide RNA; ii) transposon subunit, which includes the “adaptor protein” TniQ, transposase TnsB, and AAA+ATPase TnsC that is believed to communicate between transposase and targeting proteins;^[^ ^68^ ^]^ of note, type I also contains TnsA, which could form complexes with TnsB; and iii) donor DNA stored in transposons. The mechanism of CAST is depicted schematically in Figure 4c. Although more detailed molecular assembly and target recognition mechanisms of the system have been elucidated,^[^ ^69^ ^]^ we will not discuss them in this review due to space limitations. Type‐I CAST systems contain many more complex components. Although it has a low off‐target rate and high precision, its insertion lacks directional properties, which substantially limits its applications. In addition, although the V‐K CAST system is relatively simple, its high off‐target rate limits its use. Moreover, it often produces many post‐editing byproducts, frequently accompanied by plasmid backbone insertion, related to the absence of TnsA to cleave both ends of the donor DNA.^[^ ^66^ ^]^ Therefore, based on the V‐K system, TnsB has been fused with an incision‐homing endonuclease, to develop the HELIX directional insertion tool, which combines the advantages of the two CAST systems.^[^ ^70^ ^]^ The future direction of CAST is predicted to be based on sensible modifications and truncations of structure and function, aiming to reduce the risk of orthogonal reactivity and extend CAST to more complex application scenarios.

Transposon‐based tools offer certain benefits including minimized DSBs, large cargo capacity, and great programmability. However, they are limited by safety considerations, including the lack of specificity, off‐target effects, and interactions with in vivo transposons. Utilizing transposases with enhanced target selectivity or employing CRISPR elements to guide transposable elements are effective strategies for reducing random insertions and off‐target events.^[^ ^71^ ^]^ In addition, a new class of noncoding RNA called bridge RNA has been reported through systematic analysis. Bridge RNA could specifically bind to certain transposases, recognize donor and target DNA, and ultimately facilitate the precise recombination of large DNA fragments.^[^ ^72^ ^]^ Furthermore, the genomes of several organisms, including humans, contain a large number of unknown “dormant” transposons that cannot transpose autonomously. Exogenous transposases are most likely to interact with endogenous transposons, potentially causing abnormal activation and additional transposition events, which could potentially lead to genotoxicity.^[^ ^73^ ^]^ Overall, as natural gene‐moving elements, transposons have a significant advantage in terms of efficiency, enabling the efficient knock‐in of large fragments; however, as the current cognition of transposons is still superficial, transposons are of complex physiological and pathological relevance. Therefore, there is a need for long‐term safety assessments before further application of transposons in mammalian cells.

### 3.3 Integrase

**Figure 5:**
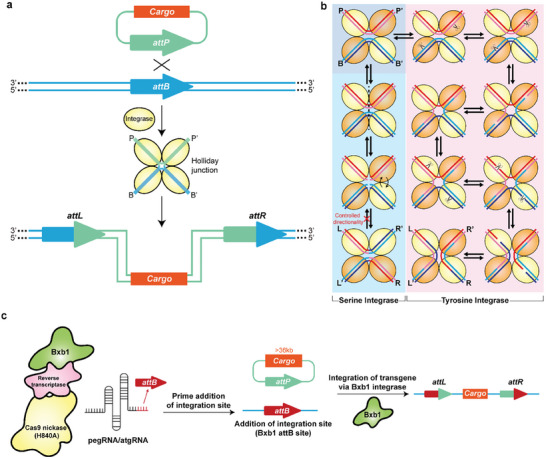
Integrase‐mediated Gene Insertion. a) The mechanism of Integrase‐mediated gene insertion. The integrase binds to two sites. The two int‐bind sites are paired to form a synaptic complex with a juxtaposition cross‐site. The recombinase then catalyzes DNA cleavage, strand exchange, and reconnection in the synaptic complex. Finally, the synaptic complex decomposes and releases the product.^[^ ^75^ ^]^ b) Strand exchange process mediated by serine integrase and tyrosine integrase. Serine integrase cleaves all strands once for strand exchange driven by the position exchange of protomer, while tyrosine integrase first realizes the exchange of single strands through a pair of subunits (orange), and then completes the exchange of another pair of single strands through another pair of subunits (yellow). Every step of the TI‐mediated recombination process is reversible, while the SI‐mediated last step has controlled directionality. This controlled directionality and a simpler recombination mechanism make SI more efficient than TI.^[^ ^75^ ^]^ c) The mechanism of PASTE. The CRISPR‐Cas system could insert genes at any site but the length of the edited fragment is limited, and the integrase could insert long‐fragment genes accurately and efficiently, but its highly conserved recognition site limits its application flexibility. The PASTE uses H840A‐Cas9 nickase (nCas9) and reverse transcriptase to introduce an integrase site (attB) on the genomic site, and then uses Bxb1 for long fragment gene insertion. The system combines the flexibility of the CRISPR‐Cas system and the high cargo capacity and accuracy of the integrase.^[^ ^49^ ^]^

Integrase, or site‐specific recombinase, recognizes specific genomic sites for targeted gene recombination. This process of site‐specific recombination can be summarized as follows (**Figure** **5**a): i) two integrase molecules bind to two recombination sites; ii) paired integrase binding sites form juxtaposed synaptic complexes; iii) in the complex, integrases catalyze DNA breakage, strand exchange, and reconnection; finally, iv) the synaptic complex disassembles and releases the recombinant product. Integrase is widely distributed, with examples such as Cas1‐Cas2 involved in forming immune memory in bacteria and archaea;^[^ ^74^ ^]^ and Cre, λint, and φC31, which participate in viral gene integration and replication. Phage‐encoded integrases, owing to their large cargo capacities, have become integral members of gene‐insertion toolkit.

Phage‐encoded integrases fall into two groups based on their sequence homology and mechanisms: tyrosine integrase (TI) and serine integrase (SI).^[^ ^75^ ^]^ TI binds to inverted repeat sequences and combines the two sites together in the form of a tetrameric complex or synapse. The formation of the correct synaptic complex is necessary to activate the recombinase. The catalytic tyrosine residues in the two recombinant enzyme monomers in the tetramer attack the DNA backbone, resulting in a covalent 3′ phosphotyrosine intermediate and a free 5′–OH terminal DNA strand. The free ─OH group from the opposite DNA substrate then replaces the covalently bound integrase, creating a recombinant joint resembling a Holliday junction.^[^ ^76^ ^]^ These steps are repeated for the remaining two recombinase monomers and DNA strands to resolve the structure and complete DNA exchange. The recombinant substrates of TI usually comprise inverted repeats,^[^ ^77^ ^]^ and TIs could be further divided into two subfamilies: i) integrases that recognize a 240‐bp phage attachment site (attP) and a 21‐bp bacterial host attachment site (attB) (e.g., λInt) and ii) those that could recognize two 34‐bp loxP sites (e.g., Cre). λInt recognizes the attP and attB for integration reactions, producing the protophage and forming new attachment sites attL and attR on both sides. At the same time, the recombination site of Cre, loxP, is much simpler than that of the λ model. The loxP is a special sequence with 13‐bp reverse repeats at both ends and directionality,^[^ ^75^ ^]^ which exists in the genomes of both phages and bacterial hosts. Besides varying recognition sites, these groups mainly differ in their requirements for host proteins.^[^ ^76^ ^]^ The activity of λInt requires host proteins like IHF, making its recognition sites larger, more complex, and inclusive of the binding sites of integrase and accessory proteins. Generally, TI provides simplicity and speed in relatively straightforward gene editing and serves as the basis for the development of the Gateway cloning method. This system utilizes precise recognition of att sites by λint, to efficiently rearrange DNA with compatible att sites on both sides, overcoming the laborious, time‐consuming, and site‐limited issues associated with traditional cloning. Its flexibility, high efficiency, and broad application range make it popular for molecular cloning.^[^ ^78^ ^]^

However, phage‐encoded TIs have several major drawbacks, particularly in straightforward cloning and DNA mobilization applications. First, because of their reliance on host‐encoded helper proteins, TIs must recognize an att site that accommodates multiple integrase and helper protein‐binding sites, resulting in a long and complex site (up to 200 bp). Additionally, long att sequences (attP, attL, and attR) and host protein requirements limit the cross‐species/generic use of TIs.^[^ ^79^ ^]^ Moreover, TIs show robust integration activity at supercoiled attP sites and potent cleavage activity at attL and attR sites, with the involvement of auxiliary protein(s), while other types of TIs, represented by Cre/loxP and Flp/FRT, have the issue of cleaving integrated DNA because of their simplistic recognition sites; thus, TIs mostly exhibit strong reverse reaction activity. Because of its reverse reactivity, TI‐mediated gene insertion has a lower success rate, thus making SIs a more favorable choice for gene insertion.

A key benefit of SI‐catalyzed integration is that it does not require host proteins. The integrase recognizes specific DNA sequences at the attP and attB sites and integrates phage nucleic acids into the host chromosome, by catalyzing recombination between these sites. This process features complete strand cleavage prior to strand exchange and rewiring (Figure 5b). In addition to the differences in integration mechanisms, SIs offer many advantages over TIs. Compared to redundant and complicated TI‐recognition sites, the attP and attB sites recognized by SI typically span less than 50 bp, with distinct inverted repeat sequences.^[^ ^80^ ^]^ The excision activity of SI for attL and attR sequences is extremely low, despite some systems showing slight cleavage;^[^ ^81^ ^]^ Thus, controlled directionality enhances gene stability after successful integration and improves the applicability of SI for gene insertion in heterologous organisms. The phage‐encoded recombination direction factor enables site‐directed recombination between attL and attR sequences, thereby expanding the editing range.^[^ ^81b^ ^]^ Furthermore, SIs do not require substrate supercoils, allowing efficient recombination of linear fragments in vitro integration and cleavage reactions. Due to its convenience, accuracy, and security, SIs have gradually become the preferred tool for gene integration.

Although integrases are highly efficient and moderately easy to operate in single‐gene insertion scenarios, their orthogonal use could enable multi‐segment knock‐ins in a single genome.^[^ ^82^ ^]^ A distinctive feature of integrases is their tight control over integration and cleavage, ensuring precise gene insertion. However, owing to inherent sequence limitations, integration only targets a limited number of genomic sites, restricting the flexibility of its application. Some integrases, such as φC31, may encounter chromosomal rearrangement issues between landing sites, leading to DNA damage and other severe outcomes.^[^ ^83^ ^]^ This represents another important limitation to the broader use of these enzymes in genetic engineering.

An effective strategy for addressing these limitations is integration with other engineering elements. As an integrase relies on protein‐nucleic acid interactions for targeted insertion, one approach is to pair it with a highly programmable CRISPR‐Cas system. This involves the preintroduction of integrated landing pads at sites of interest, ideally using DSB‐minimizing tools.^[^ ^84^ ^]^ Programmable addition via site‐specific targeting elements (PASTE) was designed based on these strategies (Figure 5c).^[^ ^49^ ^]^ The first step involves screening metagenomes and orthologous genomes for the SI with the highest activity and specificity, followed by fusion with PE. PASTE enables the insertion of a 36‐kb fragment with an efficiency >10%. Moreover, compared with transposase‐based gene‐insertion tools, PASTE enables more precise insertions, allowing for the design of predictable fusion gene products. However, owing to the complex processes involving DNA nicks, reverse transcription, balancing/resolution of fragments, and DNA recombination, the insertion efficiency of PASTE decreases. Nonetheless, PASTE represents a remarkable advancement in gene‐editing technologies.

Integrases are valuable tools for precise DNA rearrangement. Because of their high efficiency, convenience, and accuracy, these have been widely used for gene editing in various organisms, including higher eukaryotes. This accuracy stems from the specific recognition of landing sites. However, the specificity limits the application of integrases in certain situations, such as in mammalian cells. A plausible strategy to overcome these limitations involves screening or upgrading integrases with a high affinity for similar endogenous sequences (also known as pseudosites) or preintroducing landing pads using a programmable CRISPR‐Cas system. However, it is critical to reduce potential genotoxicity caused by DSBs during the introduction process. Undoubtedly, nature's highly diverse integrases have great developmental potential. Introducing specific landing pads at safe sites (such as genomic safe harbors) under the guidance of other programmable elements represents the primary future development trend in this field.

### 3.4 Other Insertion Tools

**Figure 6:**
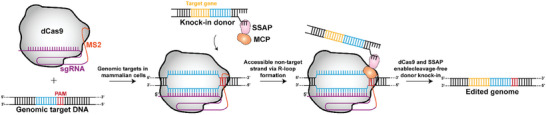
Molecular Mechanism of the dCas9‐SSAP Gene Editor. Deactivated Cas9 (dCas9) is unable to cleave nucleic acids, but could bind to the target DNA strand under the guidance of MS2‐gRNA (which consists of an RNA aptamer MS2 stem‐loop fused to a guide RNA backbone) to form an R‐loop and release the non‐target strand. SSAP fused to MS2 coat protein (MCP) specifically recognizes the MS2 stem‐loop and is recruited to the R‐loop to form a complex with dCas9‐MS2‐gRNA. Then, homologous recombination is achieved with the help of SSAP and the aim of knock‐in with minimized DSBs is achieved.^[^ ^85^ ^]^

There are other noteworthy methodologies as well in addition to the gene‐insertion tools mentioned above. For instance, the phage‐derived single‐stranded annealing protein has demonstrated potential for enhancing the insertion efficiency of long sequence edits generated by means of Cas9 cleavage.^[^ ^85^ ^]^ Notably, when paired with catalytically deactivated Cas9 (dCas9), single‐stranded annealing protein was later found to be capable of achieving precise insertion editing of exogenous sequences with comparable efficiency (**Figure** **6**). Intriguingly, deactivated Cas9‐single‐stranded annealing protein‐mediated gene editing has achieved near‐zero off‐target rates, while maintaining efficient performance in a variety of contexts, including human embryonic stem cell lines.^[^ ^85^ ^]^ Therefore, this strategy holds great promise for further investigation.

## 4 Challenges and Progress in Knock‐in Gene Therapy

Gene therapy refers to a treatment that introduces genetic material into cells to achieve therapeutic effects by supplementing or repairing damaged genes, or modifying cells to equip them with new or enhanced functions, thereby offering vast potential for the treatment of genetic diseases, combating aging, and controlling infectious diseases.^[^ ^86^ ^]^ Traditional drug development is a complex and systematic process that cannot eliminate the problems of a long cycle, high cost, and low success rate. It takes billions of dollars and 10–15 years for the development of an innovative drug. In contrast, gene therapy requires less time and cost because of its excellent programmability.^[^ ^87^ ^]^

On November 16, 2023, the world's first CRISPR gene‐editing therapy, approved for the treatment of therapeutic sickle cell disease and transfusion‐dependent beta‐thalassemia, received conditional marketing approval from the MHRA.^[^ ^88^ ^]^ This was a landmark moment for gene therapy, and the development of gene knock‐in could substantially broaden the indications for gene therapy. Gene knock‐in therapy extends beyond providing effective copies of functional genes and could introduce exogenous therapeutic genes and other functionalities. In particular, the precise insertion of lengthier fragments holds immense promise for clinical applications.

**Figure 7:**
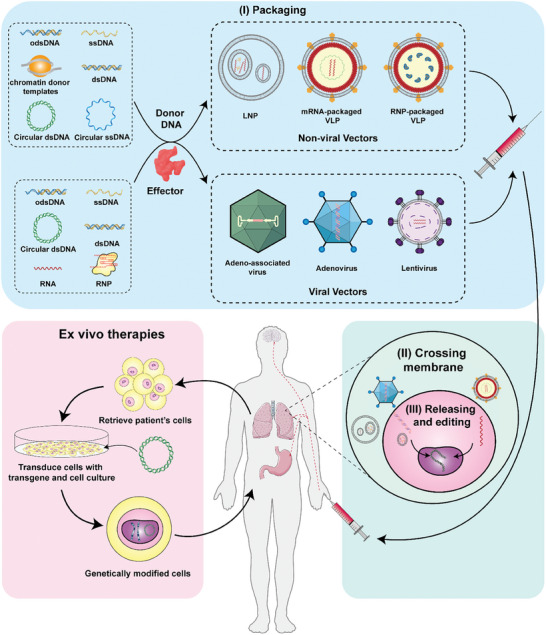
Overview of the Process and Progress of Therapeutic Gene Knock‐in. Gene therapy encompasses both ex vivo cell editing followed by reinfusion and the delivery of editing agents in vivo for direct functionality.^[^ ^89^ ^]^ The biggest challenge currently faced by gene insertion lies in achieving breakthroughs and wide use in in vivo studies. In an ideal in vivo knock‐in treatment process, both donor DNA and effector molecules could be rationally encapsulated within carriers in various forms. These carriers are capable of traversing multiple physiological barriers to reach the target tissue or cells and release their contents. Ultimately, the delivered content facilitates the knock‐in of the donor DNA into the predetermined genomic location. Parts of the figure were drawn by using pictures from Servier Medical Art. Servier Medical Art by Servier is licensed under a Creative Commons Attribution 3.0 Unported License (https://creativecommons.org/licenses/by/3.0/).

Currently, most gene therapy procedures are conducted ex vivo.^[^ ^89^ ^]^ For instance, certain cells such as hematopoietic stem cells are isolated from the human body, genetically modified ex vivo, and then reimplanted. However, most cells do not satisfy the prerequisites for ex vivo modification, underscoring the need for efficient in vivo editing techniques, particularly gene knock‐in procedures.^[^ ^89^, ^90^ ^]^ In contrast to other forms of genetic modification, gene insertion necessitates the delivery of donor DNA. This requirement imposes challenges on the loading capacity of vectors, as well as the selection and generation of suitable donor templates. Generally, the therapeutic process of gene knock‐in could be divided into three stages: i) assembly of carriers and cargo, ii) assembly of physiological barriers, and iii) disassembly and release of editing tools (**Figure** **7**). Recent studies have optimized several improvements in these stages, to enhance the efficiency and safety of gene knock‐in.

### 4.1 Assembly of Carriers and Cargos

The assembly of carriers and payloads is a crucial step in determining the success of the entire gene knock‐in process. Existing carriers have difficulty in balancing two important evaluation indicators: safety and payload capacity. The requirement for donor DNA in gene knock‐ins (especially for larger therapeutic genes) increases the burden. This problem could be easily resolved ex vivo through co‐transfection, electroporation, and other technical methods. However, these methods are not applicable to in vivo treatment. In addition, the assembly process is considerably influenced by gene knock‐in tools, carrier genes, and carrier selection. Although we have delved deeply into gene knock‐in tools, it must be pointed out that the form and modification of the cargo gene have a profound impact on the efficiency of the gene knock‐in process, as discussed in detail below. The choice of carriers for overcoming physiological barriers is discussed in the section.

In comparison to other editing approaches, the form, chemical modifications, and design methodology of the donor DNA exert a considerable influence on the efficiency of the knock‐in process (**Table** **2**). Historically, dsDNA has been widely used as a template for homologous recombination. However, dsDNA is susceptible to exploitation via the NHEJ repair pathway, leading to significant cytotoxicity and numerous unexpected products. Single‐stranded DNA (ssDNA) donors have somewhat mitigated these drawbacks; they exhibit superior knock‐in efficiency and reduced cytotoxicity or anticipated alterations. However, ssDNA is time‐consuming and expensive to generate, hindering its widespread use. A promising innovation involves the utilization of dsDNA with overhangs, effectively integrating the advantages of both dsDNA and ssDNA; this strategy exhibits higher efficiency and safety, enabling synthesis through straightforward steps. Plasmids, chromatin DNA, and circular ssDNA are common donor types that require further development.

**Table 2:** Strategies and summaries of donors in gene knock‐in.

Donor type		Structural specificity	Knock‐in efficiency (based HDR)	Production difficulty	Off‐target / Potential toxicity	Cargo	References
dsDNA	classic linear dsDNA	The end could be chemically modified	>25% in human T cells <10% in human RPE1 cells	+	+++	>10 kb	[93, 137]
	circle dsDNA (plasmid)	‐	>30% in human T cells	+	+++	>10 kb	[93]
	Overhang dsDNA (odsDNA)	3′ overhang	>4‐folds of classic dsDNA	+++	+	>2.5 kb	[138]
	chromatin‐form plasmid	Plasmid entanglement on histones (chromatin reconstituted by salt dialysis)	>2‐folds of plasmid	+++	+	>700 bp	[139]
ssDNA	short ssDNA (sssDNA)	The end could be chemically modified	≈10% in HEK293T cells	+	++	<200 bp	[140]
	long ssDNA (lssDNA, >200 bp)	The end could be chemically modified	≈12% in TLR‐MCV1 cells >15% reported in Zebrafish	+++	++	<4 kb	[141]
	circle ssDNA (cssDNA)	‐	>1.2‐folds of lssDNA	++	++	>10 kb	[141a]
	hybrid ssDNA	Special structure at the end	>2‐folds of classic dsDNA	+++	+	>2 kb	[91]

Various strategies have been explored to improve the knock‐in efficiency of donor DNA, mainly including the introduction of functional sequences and modifications to donor DNA, in addition to altering the donor's composition. One effective approach involves appending a Cas9 target sequence, including a gRNA target and PAM, to the end of the template, which enhances donor DNA utilization and subsequently improves knock‐in efficiency.^[^ ^91^ ^]^ Moreover, appropriate chemical modifications at the donor end could effectively increase the editing level.^[^ ^92^ ^]^ Notably, strategies such as adjusting the ratio of homologous arms to cargo genes and leveraging transcriptional coupling have demonstrated promising potential.^[^ ^92^, ^93^ ^]^ In essence, a judicious combination of suitable donor types and enhanced strategies could facilitate more precise and safe clinical treatments.

### 4.2 Crossing the Physiological Barriers

Gene knock‐in tools in the form of DNA, mRNA, or ribonucleoprotein particles must overcome numerous physiological barriers before entering the target; the carrier plays a critical role in this trajectory. An effective intrabody vehicle should encapsulate and safeguard its cargo against degradation or disruption, navigate through cellular membranes and other barriers to access the cell interior, and ensure targeted delivery to specific subcellular compartments. Subsequently, the payload should be released within suitable subcellular compartments. Currently, primary delivery vehicles can be divided into viral and nonviral vectors, each with distinct application benefits (**Table** **3**).^[^ ^94^ ^]^

**Table 3:** Examples of commonly used gene insertion vectors and delivery systems.

Name	Cargo	Immunogenicity	Expression level	Expression duration	Advantages	Challenges	References
Adeno‐associated virus	<4.7 kb	‐	+++	>6 months	1. Good security 2. Highly biocompatible 3. Multiple wild serotypes could target different tissues 4. Strong diffusion in various tissues and organs	1. Poor ability to carry DNA 2. Risk of off‐target editing caused by persistent cargo expression 3. Risk of viral genome integration 4. Low expression levels in vitro	[95, 98, 101, 102, 116]
Lentivirus	<10 kb ssRNA	+	++	Long‐term stable expression	Large transport capacity	1. Potential genotoxicity 2. Risk of off‐target editing caused by persistent cargo expression 3. Risk of viral genome integration 4. The cost of acquiring sufficient titers is high	[104, 105, 106, 107]
Adenovirus	<7.5 kb dsDNA	++	+++	≈3 weeks	1. Large transport capacity 2. Good biological and genetic stability 3. High transduction efficiency 4. Low production cost	1. Leading to T‐cell‐mediated cytotoxicity 2. Risk of off‐target editing caused by persistent cargo expression	[100, 108, 109, 110]
Lipid nanoparticles	>5 kb mRNA or DNA or sgRNA	–	+	<1 week	1. Excellent biocompatibility, almost non‐immunogenic and cytotoxic 2. Different cargos could be delivered simultaneously (e.g., mRNA and sgRNA for Cas9 expression) 3. Properties of LNP (e.g., particle size, surface properties) could be easily adjusted by changing components. 4. LNP manufacturing for large‐scale production has been demonstrated to be feasible	1. Large adverse effects with intravenous administration 2. Difficult to target nonhepatic tissue	[112]
Virus‐like particle	mRNA or protein or RNP	‐	+	≈72 h	1. Good security 2. Good targeting 3. High transfection efficiency	1. Low success rate of in vivo editing 2. Costly 3. Integration risks	[89, 117]

In the realm of viral vectors, the adeno‐associated virus (AAV) is widely adopted due to its high safety profile and superior programmability, with many AAV‐based therapies already approved by the US Food and Drug Administration.^[^ ^95^ ^]^ Naturally occurring AAVs display various serotypes and variants, with distinct capsid proteins offering different tissue‐specific targeting capabilities. For instance, AAV8 is typically used for liver targeting,^[^ ^96^ ^]^ whereas AAV9 is preferred for cardiac targeting.^[^ ^97^ ^]^ Techniques such as capsid engineering could be used to design customized AAV variants targeting an even broader spectrum of tissues or cells.^[^ ^98^ ^]^ One major restriction in AAV development pertains to its limited payload capacity (<4.7 kb), which is further diminished by the need to encode promoters and *cis*‐regulatory elements. To mitigate this, strategies involving dual AAV packaging and smaller effector proteins have been adopted.^[^ ^99^ ^]^ However, for in vivo gene knock‐in, the effective payload capacity of AAV is still far from sufficient, and there is a need to explore strategies for increasing the payload capacity.

One concern with AAV vectors is their potential for sustained gene expression, lasting several years, inherently increasing the risk of off‐target editing.^[^ ^100^ ^]^ Furthermore, prolonged expression of effector proteins, such as Cas9, may trigger ongoing immune responses, leading to targeted destruction of edited cells by the immune system.^[^ ^101^ ^]^ The persistent expression could be addressed through methods including small‐molecule compound‐based regulation, co‐expression of specific proteases, expression in response to miRNA concentration, and other means of degradation or silence effectors,^[^ ^98^, ^102^ ^]^ thus preventing over‐editing. Limiting the gene editor payload expression to specific tissues could decrease the likelihood of off‐target editing in non‐target tissues. Overall, the spatial and temporal control of AAV‐delivered gene‐editing tools could optimally enhance the specificity and safety of gene editing.

Despite numerous studies on potential transduction pathways of certain AAV serotypes, a complete understanding of the in vivo kinetic processes of AAVs still remains elusive and it is not known whether significant differences exist between the kinetic processes of different serotypes. A deeper understanding of the transduction mechanisms of AAVs will aid in designing safer and more effective AAVs with high target specificity.

Lentiviral vectors (LVs), stemming from the human immunodeficiency virus, encapsulate a single‐strand positive‐sense RNA genome that transcribes into DNA and upon nucleus entry, integrates semi‐randomly into the target cell's genome.^[^ ^103^ ^]^ While this semi‐random integration could be beneficial under certain circumstances, it often leads to unpredictable genotoxicity and immunogenicity, necessitating integrase‐defective LVs.^[^ ^104^ ^]^ The primary advantage of LVs lies in their large payload capacity (≈10 kb), permitting them to carry substantial knock‐in tools such as PE or multiple guide RNAs for multi‐site editing.^[^ ^105^ ^]^ Moreover, LVs showcase impressive delivery efficiency in both dividing and nondividing cells.^[^ ^106^ ^]^ Despite these features, LVs are infrequently used for in vivo editing, primarily due to their potential for genomic integration. Although theoretically lacking integration capability, integrase‐defective lentiviral vectors do not completely eliminate the possibility of integration into the human genome, implying potential risks from the sustained expression of the editing tool.^[^ ^107^ ^]^ In summary, the genotoxicity, immunogenicity, and high production costs associated with LVs pose considerable obstacles to their clinical application.

Adenoviral vectors (Ads) are among the most popular vectors in gene therapy because of their broad host cell range and high infection efficiency.^[^ ^108^ ^]^ Under ideal transduction conditions, the transduction efficiency of Ads could reach up to 99%.^[^ ^109^ ^]^ Additionally, the biological characteristics of Ads are well studied; they rarely integrate into the host genome and exhibit genetic stability with minimal gene mutations. Hence, Ads are highly suitable for transient gene expression. Like AAVs, Ads also have multiple serotypes, each displaying different tissue or cell specificity, enabling researchers to select the appropriate serotype based on clinical needs.^[^ ^108^ ^]^ Importantly, Ads could be industrially produced at large scales and high titers, implying reduced injection volumes, a considerable advantage in animal experiments and clinical trials.^[^ ^108^ ^]^ However, owing to its high immunogenicity and adjuvant‐like properties, using Ad as a vector might lead to the production of neutralizing antibodies against effector proteins and trigger T cell‐mediated cytotoxicity.^[^ ^110^ ^]^ Although reducing the viral antigen expression could lower their immunogenicity,^[^ ^108^ ^]^ the wider application of Ads‐mediated in vivo gene editing requires further research and engineering.

Currently, viral vectors are being rapidly developed and widely used. However, immunogenicity, sustained payload gene expression, off‐target possibilities, potential for genome integration, and manufacturing costs limit their clinical applications. In contrast, owing to their unique advantages, nonviral vectors offer promising prospects for future development.

The outbreak of COVID‐19 has put mRNA vaccines based on lipid nanoparticle (LNP) vectors in the spotlight.^[^ ^111^ ^]^ For decades, artificially manufactured LNPs have been used to deliver nucleic acid payloads, including small interfering RNA and therapeutic mRNA,^[^ ^112^ ^]^ and could be designed to possess different pharmacokinetic characteristics and tissue specificity, by adjusting their components.^[^ ^112b^ ^]^ LNP‐based delivery results in transient expression of therapeutic genes, which minimizes the likelihood of off‐target editing and reduces the chance of target cells being recognized and attacked by the immune system, thus enabling target cells to survive for an extended period.^[^ ^113^ ^]^ Notably, since LNPs are easily enveloped by apolipoproteins like APOE, most injected LNPs accumulate in the liver.^[^ ^112b^ ^]^ Therefore, while LNPs are most commonly used for delivering therapeutic substances to the liver; this characteristic limits their use in nonhepatic delivery. Numerous studies have suggested that it is feasible to adjust the multi‐tissue targeting ability of LNPs through strategies such as local injection,^[^ ^114^ ^]^ changing or adding LNP components, embedding antibodies corresponding to target tissues on the LNP surface,^[^ ^115^ ^]^ or suppressing editing levels in the liver, thereby achieving delivery to non‐hepatic tissues such as the eyes, inner ears, spleen, and lungs.

In addition to LNPs, virus‐like particles (VLPs) are commonly used as non‐viral vectors. VLPs are highly structured particles formed by the self‐assembly of one or more viral structural proteins. They bear a high degree of morphological consistency with their corresponding natural viruses but lack viral genetic material. This feature reduces the potential risk associated with viral vector self‐replication and modulates VLP cell‐target specificity through modified envelope glycoproteins. Notably, VLPs are large in size, allowing for transient delivery of gene editors in various forms, like DNA, mRNA, or ribonucleoprotein particles.^[^ ^116^ ^]^ Delivering of ribonucleoprotein particles does not involve long‐term gene expression, thus minimizing the risk for off‐target editing.^[^ ^117^ ^]^ However, since VLPs are based on a viral backbone, their immunogenicity and low in vivo efficiency have been a matter of wide concern.^[^ ^89^ ^]^ Recently, Zhang et al. reported that PEG10 could be programmed to package desired mRNA payloads and Cas9 nucleases.^[^ ^118^ ^]^ As a mammalian retrovirus‐like protein, it uses an endogenous mammalian protein scaffold, significantly reducing immunogenicity, as compared with that observed upon use of retrovirus VLPs, while improving efficiency and safety.

The development of gene therapy drug delivery carriers is entering a new stage. In addition to mainstream viral and nonviral carriers, novel carriers such as exosomes and gold nanoparticles have shown unique potential.^[^ ^119^ ^]^ However, to date, only a few in vivo gene knock‐in tools have been used in clinical trials. The main concern in their application is the difficulty of effectively encapsulating effector molecules and donor DNA and achieving a relatively high local concentration. Moreover, there is an urgent need to resolve safety issues such as genotoxicity and off‐target editing caused by long‐term in vivo expression of editing tools.

### 4.3 Content Releasing and Targeting of the Genome

Upon ideal delivery of the knock‐in tool and donor DNA into a predetermined subcellular organelle, the content is systematically released and guided to distinct genomic locations by means of various matching methods. However, imprecise matching could result in unintended editing, which is commonly referred to as off‐target editing. Although the mechanisms underlying off‐target editing have been extensively investigated,^[^ ^120^ ^]^ this challenge still requires a solution. Notably, off‐target editing could induce silencing of functional genes, elimination of large gene fragments, and even chromosome deletions. Therefore, for the clinical application of knock‐in therapy, it is imperative to circumvent or anticipate the potential genotoxicity caused by off‐targeting.

For knock‐in procedures employing nucleases, such as Cas9, an effective strategy involves the use of nCas9 with exclusive single‐stranded nuclease activity or deactivated Cas9 devoid of nuclease activity. Alternatively, optimizing and modifying guide RNA could mitigate off‐target events.^[^ ^121^ ^]^ In research contexts utilizing integrases, transposases, and similar tools, the primary investigative emphasis shifts toward the delineation of more precise subtypes of recognition sites, accomplished through methods such as screening and directed evolution. The use of deep learning and artificial intelligence to develop high‐throughput off‐target assessment platforms shows considerable promise.^[^ ^122^ ^]^ The primary evaluation determinant of these platforms is free‐binding energy. A deeper understanding of the off‐target mechanisms would likely enhance the precision of these assessment methods, thus promoting advancements in gene therapy.

## 5 Some Attempts Aimed at Application of Gene Knock‐In in Disease Treatment

So far, although knock‐in is rarely used for clinical treatment of relevant diseases, its ability to modify genes has gradually shown great potential, which includes modification of immune cells to better kill tumor cells and modification of xenogeneic organs to escape the clarity of the immune system. In fact, the potential for programmable modifications goes far beyond this, with curative potential for inflammation, cancer, and so on.

### 5.1 Cytotherapy

Despite substantial advancements in biomedicine, cancer remains an affliction with limited effective treatments and high mortality rates. Traditional therapies, such as radiotherapy and chemotherapy, are beneficial for certain malignant tumors but are associated with substantial side effects, poor patient tolerance, and other shortcomings. However, cellular immunotherapy offers a promising alternative.^[^ ^123^ ^]^ It effectively mitigates these drawbacks and boasts precise targeting properties largely attributable to the critical role of gene knock‐in, which enables diverse genetic modifications in the extracted cells.

Indeed, chimeric antigen receptor T‐cell therapy exemplifies a groundbreaking innovation in cellular therapy.^[^ ^124^ ^]^ The cornerstone of chimeric antigen receptor T‐cell therapy is the stable expression of chimeric antigen receptors on T cells, traditionally achieved through the inherent integration of viral vectors. Nevertheless, this semi‐random gene modification approach carries uncontrollable risks, such as carcinogenesis. Consequently, there is a trend to explore the use of CRISPR‐Cas and transposases for targeted knock‐in methods, aiming for safer and more controlled chimeric antigen receptor T‐cell production, which has immense application potential.^[^ ^125^ ^]^

In line with this, Editas Medicine introduced a novel cellular therapy named EDIT‐202 in 2022. Its core technology involves knocking genes such as mbIL‐15 and CD16 into induced pluripotent stem cells using their proprietary SLEEK gene knock‐in platform, followed by the differentiation of these stem cells into natural killer cells.^[^ ^126^ ^]^ These genetically edited natural killer cells demonstrated formidable tumor‐killing potency, addressing the treatment needs for specific malignant cancers.^[^ ^126^ ^]^ As we continue to refine gene knock‐in techniques, we could expect to further enhance the safety and efficacy of cell engineering, potentially paving the way toward a complete cure for cancer.

### 5.2 Xenotransplantation

Although organ donations are increasing annually, the persistent shortage of organs remains a pressing concern.^[^ ^127^ ^]^ Allogeneic organ transplantation is a plausible solution to this problem. Since the early 20th century, humankind has experimented with transplanting couldine kidneys into humans, subsequently discovering that porcine organs, due to their size and physiological similarities to human organs, could serve as potential alternatives for individuals suffering from organ failure.^[^ ^128^ ^]^ However, the prospect of porcine organ utilization is constrained by potential immune responses and associated disease risks, including cross‐species contamination by porcine endogenous retroviruses.^[^ ^129^ ^]^

The advent of gene editing holds promise for enhancing the safety of porcine organs, with gene knock‐in effectively reducing their immunogenicity. Recent reports have documented the survival of genetically engineered pig kidneys for over two years post‐transplantation into crab‐eating monkeys when combined with immunosuppressive therapy.^[^ ^130^ ^]^ The investigators utilized Yucatan minipigs as donors and knocked in seven human genes including a human cluster of differentiation CD46 and CD55, along with a host of genes associated with transplantation risk. This intervention drastically improved the survival rate of the recipient subjects, as compared with that observed in their counterparts without the knocked‐in genes.^[^ ^130^ ^]^ Notably, the world's second patient with a genetically modified pig heart transplant died 40 days after surgery, with immune rejection issues remaining a major cause.^[^ ^131^ ^]^ As our understanding of gene knock‐ins deepens, the “masquerading” of xenogeneic and allogeneic organs will become increasingly feasible, enabling evasion of the immune system and substantially augmenting the quality of life for organ recipients.

## 6 Discussion

The emergence of precision genome‐editing technology has empowered us to manipulate human elements, from macro to micro. Unlike other forms of gene editing such as knockouts, gene insertion offers unique advantages for both basic research and practical applications. Substantial progress has been achieved in this area. SDNs are highly programmable and have been proven instrumental for efficient gene insertion across various cell lines. However, their dependence on in vivo gene repair pathways limits the extent of their insertion, and nuclease‐induced DSBs may trigger genotoxicity. Hence, DSB‐minimized tools have been developed for more secure utilization.

PE has attracted interest because of its ability to operate without donor DNA and reduce the formation of DSBs. However, PE faces challenges with respect to the insertion length. This has led to focused improvements aimed at increasing the allowable insertion length. Concurrently, natural gene transfer elements, such as transposons and integrases, which are capable of high cargo gene loads, have considerable potential. Generally, integrases exhibit superior site specificity and produce fewer byproducts, whereas the primary limitation of transposases is their weak specificity, which triggers extensive random insertions in the genome, necessitating improved targeting capability. The strict recognition of integrases creates multiple blind editing spots. Combining this approach with the CRISPR‐Cas system could effectively mitigate this issue. These enhancements epitomize the evolving prospects of gene‐insertion techniques and signal a promising trajectory for genetic engineering.

Beyond the judicious selection of gene knock‐in tools, it is crucial to optimize and engineer advancements in donor DNA and vectors, grounded in an understanding of the complete in vivo life cycle of gene knock‐ins, for improving clinical outcomes. These factors considerably influence the overall efficiency of the knock‐in process, immune response, and tissue or cell specificity. In addition, a comprehensive understanding of the off‐target effects of gene‐editing tools will facilitate the design of more effective gene therapy regimens. In particular, artificial intelligence could provide valuable assistance in predicting and assessing the potential risk for off‐target editing, which is essential for controlling the overall treatment regimen.

Choosing an appropriate site is critical for gene insertion. In clinical treatment, a safe gene harbor is most commonly used to ensure that the inserted gene does not induce hazardous changes in the cell genome. Given the multi‐factorial regulation of gene expression, it is imperative to verify whether the insertion site could ensure the efficient and stable expression of cargo genes, which requires further screening and validation.

Gene knock‐in has shown unique flexibility and complexity in comparison to other editing techniques. This is due to the fact that knock‐in could not only repair and replace non‐functional genes but also introduce exogenous genes for modification. However, current technological limitations have restricted most of its applications to in vitro settings. A comprehensive analysis of the factors influencing gene knock‐in and targeted optimization will unleash the potential of gene knock‐in in treating diseases, particularly genetic disorders. Notably, next‐generation knock‐in tools will significantly reduce the time and R&D costs associated with creating animal models, helping to address the current challenges of long drug development cycles and high costs.

Although the clinical application of gene knock‐in has sparked considerable interest, additional research is needed to precisely regulate gene expression and predict the potential adverse events triggered by the expression product. Insertion of therapeutic genes into individuals lacking certain childhood genes could potentially induce immune responses. Future efforts should focus on integrating a complete gene expression system comprising therapeutic genes and their regulatory elements, which could be regulated according to the actual expression levels or halted if endangering the individual's life and health. Notably, fine‐tuning gene regulation may encourage genetic enhancement, enabling modifications to physical traits and intelligence. The ethical implications and social risks associated with opening this Pandora's box warrant broader and more profound discussion.

Advancements in lengthening insertional fragments and refining gene knock‐in tools have enabled free manipulation of the genome. With the continuous discovery of natural tools and breakthroughs in bioengineering, gene insertion has the potential to unlock a wide range of applications, and unprecedented progress is anticipated in this field of genetic engineering.

## Conflict of Interest

The authors declare no conflict of interest.

## Author Contributions

Y.L. and J.K. contributed equally to this work.
